# Perhalogenated pyrimidine scaffolds. Reactions of 5-chloro-2,4,6-trifluoropyrimidine with nitrogen centred nucleophiles

**DOI:** 10.3762/bjoc.4.22

**Published:** 2008-07-01

**Authors:** Emma L Parks, Graham Sandford, John A Christopher, David D Miller

**Affiliations:** 1 Department of Chemistry, University of Durham, South Road, Durham, DH1 3LE, U.K.; 2 GlaxoSmithKline R&D, Medicines Research Centre, Gunnels Wood Road, Stevenage, Hertfordshire SG1 2NY, U.K.

**Keywords:** pyrimidine, rapid analogue synthesis, perfluoroheteroaromatic, nucleophilic aromatic substitution

## Abstract

**Background:**

Highly functionalised pyrimidine derivatives are of great importance to the life-science industries and there exists a need for efficient synthetic methodology that allows the synthesis of polysubstituted pyrimidine derivatives that are regioselective in all stages to meet the demands of RAS techniques for applications in parallel synthesis. 5-Chloro-2,4,6-trifluoropyrimidine may be used as a scaffold for the synthesis of polyfunctional pyrimidine systems if sequential nucleophilic aromatic substitution processes are regioselective.

**Results:**

Use of 5-chloro-2,4,6-trifluoropyrimidine as a core scaffold for the synthesis of functionalised pyrimidine systems is assessed in reactions with a small range of nitrogen centred nucleophiles. Mixtures of products arising from nucleophilic aromatic substitution processes are formed, reflecting the activating effect of ring nitrogen and the steric influences of the chlorine atom.

**Conclusions:**

5-Chloro-2,4,6-trifluoropyrimidine is not an ideal scaffold for analogue synthesis or for multiple substitution processes because purification must be performed to remove the 2-substituted regioisomer from the mixture before further reactions can be carried out. However, 4-amino derivatives can be isolated in acceptable yields using this methodology.

## Introduction

**Figure 1:**
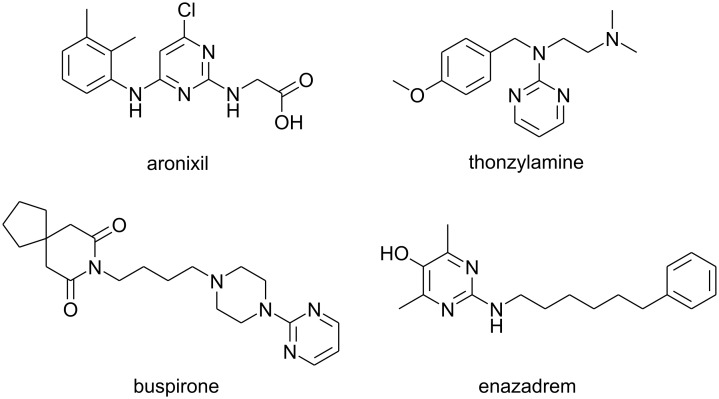
Pharmaceuticals with pyrimidine sub-units.

Highly functionalised pyrimidine derivatives are of great importance to the life-science industries and, indeed, many pyrimidine derivatives have been used for various medicinal applications (Figure 1) [1–3].

Synthesis of pyrimidine rings most commonly involves cyclocondensation reactions of amidine, guanidine or thiourea derivatives with either 1,3-diketone or 1,3-diester systems [4–5]. However, many of these reactions are not regiospecific and, furthermore, there is an added difficulty of synthesizing a range of structurally related pyrimidine analogues by parallel synthesis or rapid analogue synthesis (RAS) techniques [6–7] due to the limited range of non-cyclic polyfunctional precursors available. These limitations have, in part, provided added impetus for drug discovery programmes to develop effective synthetic methodology towards multiply substituted systems from simple readily accessible pyrimidine scaffolds [8]. Consequently, pyrimidine core scaffolds that bear multiple functionality, which may be transformed into a widely diverse range of functionalised derivatives by a sequence of efficient and regioselective reactions, are becoming increasingly important [6–7]. In particular, the attachment of amino groups to the pyrimidine nucleus by formation of carbon-nitrogen bonds is a highly desirable process but one of the most difficult to achieve in practice.

**Scheme 1:**
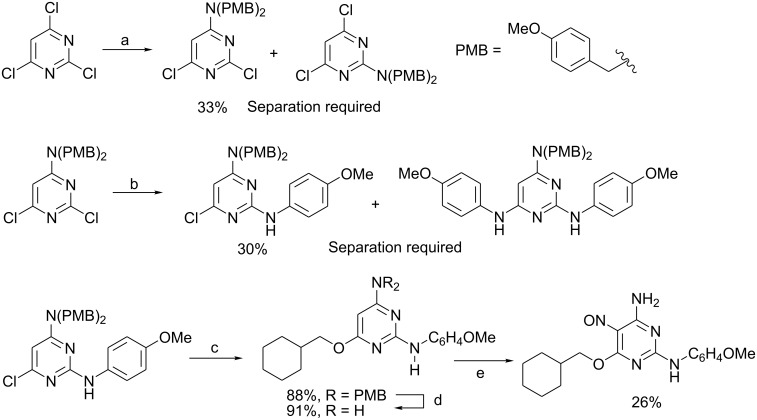
Use of 2,4,6-trichloropyrimidine as a core scaffold. *Reagents and Conditions*: a) bis(4-methoxybenzyl)amine, Et_3_N, BuOH, 75 °C; b) *p*-anisidine, Et_3_N, BuOH, DMSO, 95 °C; c) cyclohexylmethanol, Na, 170 °C; d) TFA, 60 °C; e) AcOH, H_2_O, NaNO_2_.

Pyrimidines are electron-deficient aromatic systems and, when halogenated, become very useful substrates for a variety of nucleophilic aromatic substitution (S_N_Ar) processes [9] and, since numerous chloropyrimidines are commercially available, there have been many reports of synthetic strategies concerned with creating pyrimidine-based libraries from halogenated core scaffolds. For example, recently, synthesis of an inhibitor of the cyclin-dependent kinase was developed [10] (Scheme 1) starting from 2,4,6-trichloropyrimidine as the core scaffold. However, as regioisomeric products are formed in both nucleophilic aromatic substitution stages, separation of the isomers is required after each step, making adoption of this scaffold for analogue synthesis less likely.

There remains, therefore, a requirement for efficient synthetic methodology that allows the synthesis of polysubstituted pyrimidine derivatives that are regioselective in all sequential nucleophilic aromatic substitution stages to meet the demands of RAS techniques for applications in parallel synthesis.

We are exploring the use of polyhalogenated heteroaromatic systems [11–13] as potential hetaryl core scaffolds for analogue synthesis of polyfunctional heterocyclic systems [14–16]. Polyhaloaromatic systems act as useful scaffolds because, in principle, several or all halogen atoms can be displaced by nucleophiles, giving rise to a wide range of heteroaromatic systems and, in this context, we have used a range of perfluorinated heteroaromatic molecules as synthetically versatile building blocks for the creation of new molecular scaffolds for drug discovery [14–17]. In this paper, we describe the reactivity of 5-chloro-2,4,6-trifluoropyrimidine (**1**) with a range of representative nitrogen centred nucleophiles, with the aim of exploring the regioselectivity of these nucleophilic aromatic substitution processes in order to assess the utility of the system as a scaffold for pyrimidine analogue synthesis. Whilst **1** is commercially available and has been known for some time, only a limited number of reactions have been reported (e.g. with ammonia to give the 4-amino derivative [18]) despite the fact that **1** is used widely in the fibre reactive dye industry [19]. However, a systematic study of the reactivity of this potentially valuable scaffold with other nitrogen nucleophiles has not been reported.

## Results and Discussion

A series of reactions between 5-chloro-2,4,6-trifluoropyrimidine (**1**) and a range of primary and secondary amines were carried out in acetonitrile at 0 °C in the presence of DIPEA as a hydrogen fluoride scavenger and these results are collated in Table 1. All of the reactions were monitored via ^19^F NMR and the isomer ratios measured by ^19^F NMR integration from samples taken from the reaction mixture.

**Table 1:** Reactions of amine nucleophiles with 5-chloro-2,4,6-trifluoropyrimidine (**1**).

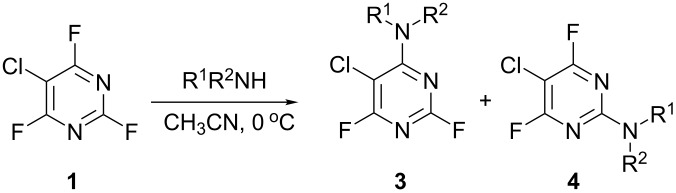			
R^1^R^2^NH	Products (isolated yield)^a^		Ratio **3** : **4** ^b^

NH_3_	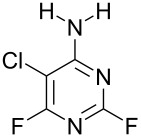 **3a**, 57%	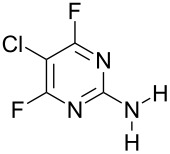 **4a**	9 : 1
EtNH_2_	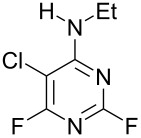 **3b**, 57%	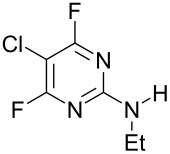 **4b**	8 : 1
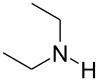	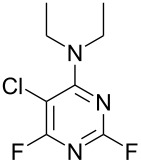 **3c**, 47%	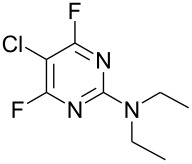 **4c**	5 : 1
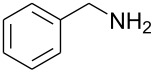	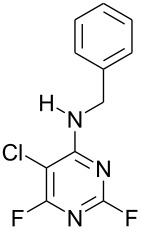 **3d**, 41 %	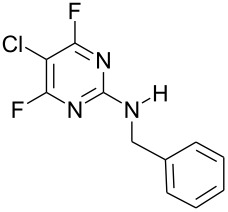 **4d**	5 : 1
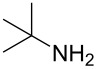	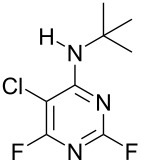 **3e**, 49%	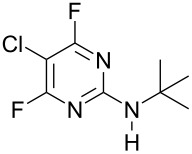 **4e**	3 : 1
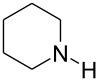	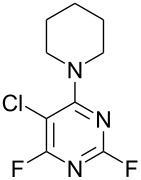 **3f**, 49%	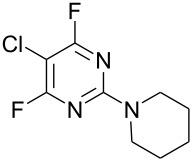 **4f**	3 : 1

^a^ Isolated yield of major products **3**. Minor products **4** were not isolated. ^b^ Ratio of **3** : **4** in crude product mixture by ^19^F NMR analysis.

Reaction of **1** with ammonia results in two isomeric products, as observed by ^19^F NMR analysis of the reaction mixture which displayed two distinctive peaks (−48.18 and −69.47 ppm) for the 4-substituted isomer and one peak (−65.44 ppm) for the 2-isomer in a 9 : 1 ratio, the chemical shifts being consistent with previous studies [18]. Similarly, reaction of **1** with ethylamine gives two isomers in an 8 : 1 ratio by ^19^F NMR as shown by the appearance of two fluorine signals (−47.48 and −70.83 ppm) and one signal (−63.59 ppm) corresponding to the 4- and 2-amino isomers respectively. Distillation afforded the 4-isomer in good yield. Other reactions gave a mixture of products which were identified by ^19^F NMR as described above and, in all cases, the major product could be isolated by either recrystallisation, or column chromatography. All products were fully characterised and ^19^F NMR analysis of the crude reaction mixtures gave the ratio of products observed.

**Scheme 2:**
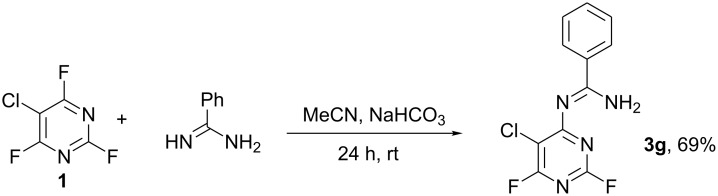
Reaction of **1** with benzamidine.

**Figure 2:**
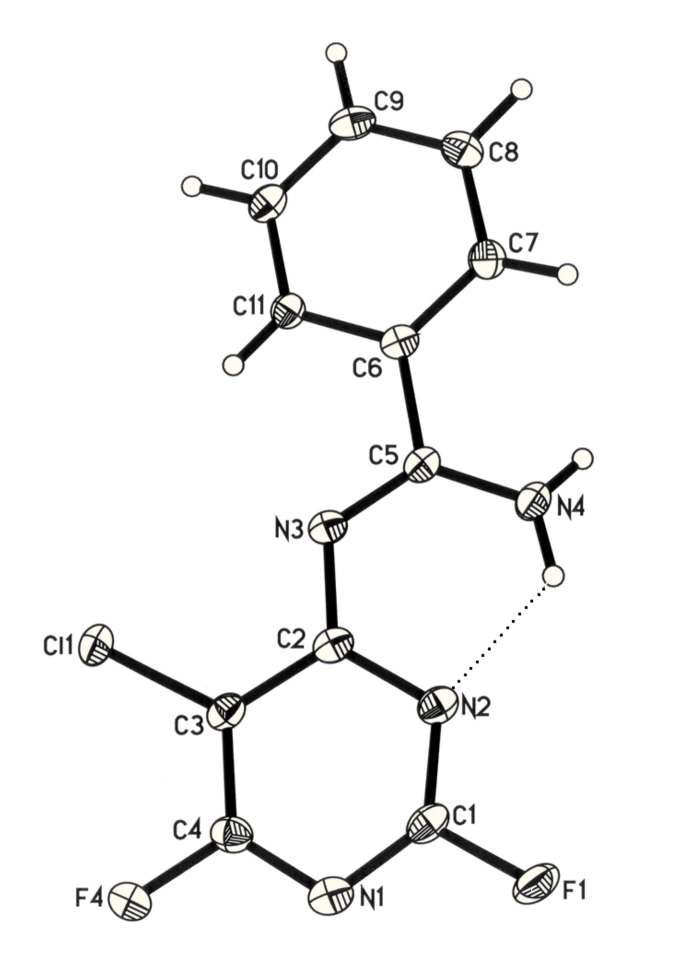
Molecular structure of **3g**.

Furthermore, when **1** was reacted with the difunctional nucleophile benzamidine, nucleophilic substitution of the fluorine at the 4- and the 2-position in a 40 : 1 ratio occurred (Scheme 2). The main product **3g** was isolated by recrystallisation from acetonitrile and characterised by X-ray crystallography (Figure 2).

In all cases, therefore, the major product obtained arises from substitution of the fluorine atom at the 4-position which is the most activated site *para* to ring nitrogen and further activated by the adjacent chlorine atom, consistent with previous observations for reactions involving perfluorinated heterocycles [13]. However, as the steric requirement of the nucleophile increases, the amount of product arising from substitution at the less activated 2-position is increased, reflecting the steric hindrance of substitution at the more activated 4-position by the larger chlorine atom located at the adjacent 5-position.

Reaction of the related 2,4,6-trifluoropyrimidine with ammonia is reported [20] to give two products in a 4 : 1 ratio and a primary amine, ethanolamine, gave a 2 : 1 ratio of products. Therefore, reactions of **1** with nitrogen centred nucleophiles are more selective than 2,4,6-trifluoropyrimidine despite the increased steric influence of the chlorine atom to nucleophilic attack. This can be rationalised by the fact that the electronegative chlorine atom activates the site *ortho* to itself towards nucleophilic attack and this partly compensates for steric factors in these reactions.

Consequently, it becomes clear that 5-chloro-2,4,6-trifluoropyrimidine (**1**) is not an ideal scaffold for analogue synthesis or for multiple substitution processes because purification must be performed to remove the 2-substituted regioisomer from the mixture before further reactions can be carried out. However, 4-amino derivatives can be isolated in acceptable yields using this methodology and, indeed, these systems could be used as scaffolds for further analogue synthesis.

## Experimental

### Typical Procedure: Synthesis of *N*-Benzyl-5-chloro-2,6-difluoropyrimidin-4-amine (3d)

A solution of 5-chloro-2,4,6-trifluoropyrimidine (0.5 g, 3 mmol), benzylamine (0.32 g, 3 mmol) and DIPEA (0.39 g, 3 mmol) in acetonitrile (50 cm^3^) was stirred at 0 °C for 2 h after which time ^19^F NMR indicated 100% conversion with the formation of *N*-benzyl-5-chloro-2,6-difluoropyrimidin-4-amine (**3d**) (−45.80 and −67.84 ppm) and *N*-benzyl-5-chloro-4,6-difluoropyrimidin-2-amine (**4d**) (−48.09 ppm) in a 5 : 1 ratio. The reaction solvent was evaporated and the crude product partitioned between DCM (3 × 40 cm^3^) and water (40 cm^3^). The organic layer was separated, dried (MgSO_4_) and evaporated to dryness to give a crude product containing **3d** and **4d** as a yellow solid (0.54 g). Recrystallisation from *n*-hexane yielded *N*-benzyl-5-chloro-2,6-difluoropyrimidin-4-amine (**3d**) (0.31 g, 41%) as a white solid; mp 57–59 °C; IR (neat, *v*, cm^−1^): 3408, 3281, 2364, 2169, 1739, 1612, 1528, 1447, 1349, 1129, 695; (Found: C, 51.7; H, 3.1; N, 16.6; C_11_H_8_ClF_2_N_3_ requires: C, 51.7; H, 3.15; N, 16.4%); δ_H_ (CDCl_3_) 4.74 (2H, d, ^2^*J*_HH_ 5.8, CH_2_), 7.39 (5H, m, Ar-H); δ_C_ (CDCl_3_) 46.2 (s, CH_2_), 93.1 (dd, ^2^*J*_CF_ 21.4, ^4^*J*_CF_ 8.0, C-5), 128.1 (s, Ar-CH), 128.4 (s, Ar-CH), 129.2 (s, Ar-CH), 136.9 (s, Ar-CH), 159.3 (dd, ^1^*J*_CF_ 222, ^3^*J*_CF_ 22.1, C-2), 162.6 (dd, ^3^*J*_CF_ 13, ^3^*J*_CF_ 5.4, C-4), 164.5 (dd, ^1^*J*_CF_ 236.2, ^3^*J*_CF_ 18.7, C-6); δ_F_ (CDCl_3_) −45.8 (1F, s, C-6), −67.9 (1F, s, C-2); *m*/*z* (EI^+^) 255 ([M]^+^, 40%), 218 (10), 178 (12).

All other experimental procedures and data are presented in Supporting Information File 1 which accompanies this paper.

## Supporting Information

File 1 Experimental procedures and data.
