# Supplementary material for: Perhalogenated pyrimidine scaffolds. Reactions of 5-chloro-2,4,6-trifluoropyrimidine with nitrogen centred nucleophiles
Source: Beilstein J Org Chem. 2008 Jul 1;4:22. doi: 10.3762/bjoc.4.22 (PMC2511021; doi:10.3762/bjoc.4.22)

**Perhalogenated pyrimidine scaffolds. Reactions of 5-chloro-2,4,6-trifluoropyrimidine with nitrogen centred nucleophiles**

Emma L. Parks^1^, Graham Sandford* ^1^, John A. Christopher^2^ and David D. Miller^2^

^1^ Department of Chemistry, University of Durham, South Road, Durham, DH1 3LE, U.K.

*^2^ GlaxoSmithKline R&D, Medicines Research Centre, Gunnels Wood Road, Stevenage, Hertfordshire SG1 2NY, U.K.*

* Corresponding author

# **Supporting Information**

# **Experimental procedures and data**

Ratios of products were calculated from ^19^F NMR analysis of the reaction mixture. All starting materials were obtained commercially (Aldrich, Lancaster or Fluorochem). All solvents were dried using either literature procedures or via the Innovative Technology solvent purification system. Column chromatography was carried out on silica gel (Merck no. 109385, particle size 0.040–0.063 mm) or using the Biotage Horizon flash chromatography system and TLC analysis was performed on silica gel or aluminium oxide TLC plates. NMR spectra were recorded in deuteriochloroform, unless otherwise stated, on a Varian VXR 500S NMR spectrometer operating at 500 MHz (^1^H NMR), 376 MHz (^19^F NMR) and 125 MHz (^13^C NMR) with trichlorofluoromethane as an internal standard (^19^F NMR). Mass spectra were recorded on a Thermo Finnigan TRACE GCMS system and a Waters Micromass LCT spectrometer. Elemental analyses were obtained on an Exeter Analytical CE-440 elemental analyser. Melting points were recorded at atmospheric pressure and are uncorrected.

**5-Chloro-2,6-difluoropyrimidin-4-amine (3a)**

A solution of 5-chloro-2,4,6-trifluoropyrimidine (0.5 g, 3 mmol) and aqueous ammonia (0.12 cm^3^) in acetonitrile (50 cm^3^) was stirred at 0 °C for 4 h after which time ^19^F NMR indicated 100% conversion with the formation of 5-chloro-2,6-difluoropyrimidin-4-amine (**3a**) (−48.18 and, −69.47 ppm) and 5-chloro-4,6-difluoropyrimidin-2-amine (**4a**) (−65.44 ppm) in a 9 : 1 ratio. The reaction solvent was evaporated and the crude product partitioned between DCM (3 x 40 cm^3^) and water (40 cm^3^). The organic layer was separated, dried (MgSO_4_) and evaporated to dryness to give a crude product containing **3a** and **4a** as a white solid (0.34 g). Recrystallisation from ethyl acetate yielded 5-chloro-2,6-difluoropyrimidin-4-amine (**3a**) (0.28 g, 57%) as a white solid; mp 161–162 °C; (Found: [MH]^+^ 165.9978; C_4_H_2_ClF_2_N_3_ requires: [MH]^+^ 165.9978); *δ*_H_ 7.96 (1H, s, NH_2_), 8.52 (1H, s, NH_2_); *δ*_C_ (acetonitrile-*d*_3_) 92.7 (dd, ^2^*J*_CF_ 39, ^4^*J*_CF_ 8, C-5), 159.4 (dd, ^1^*J*_CF_ 235, ^3^*J*_CF_ 23, C-2), 166.1 (dd, ^1^*J*_CF_ 260, ^3^*J*_CF_ 20, C-6), 165.2 (m, C-4); *δ*_F_ −46.72 (1F, s, C-6), −65.63 (1F, s, C-2); *m/z* (EI^+^) 165 ([M]^+^, 52%), 124 (4).

**5-Chloro-*N*-ethyl-2,6-difluoropyrimidin-4-amine (3b)**

A solution of 5-chloro-2,4,6-trifluoropyrimidine (0.5 g, 3 mmol) and 2.0 m ethylamine in THF (2.99 cm^3^) in acetonitrile (50 cm^3^) was stirred at 0 °C for 2 h after which time ^19^F NMR indicated 100% conversion with the formation of 5-chloro-*N*-ethyl-2,6-difluoropyrimidin-4-amine (**3b**) (−47.48 and −70.83 ppm) and 5-chloro-*N*-ethyl-4,6-difluoropyrimidin-2-amine (**4b**) (−63.59 ppm) in a 8 : 1 ratio. The reaction solvent was evaporated and the crude product partitioned between DCM (3 x 40 cm^3^) and water (40 cm^3^). The organic layer was separated, dried (MgSO_4_) and evaporated to dryness to give a crude product containing **3b** and **4b** as a yellow oil (0.54 g). Column chromatography (silica, DCM : *n*-hexane, 1 : 1) gave 5-chloro-*N*-ethyl-2,6-difluoropyrimidin-4-amine (**3b**) (0.33 g, 57%) as a white solid; mp 37–38 °C; (Found: C, 37.5; H, 3.2; N, 21.6; C_6_H_6_ClF_2_N_3_ requires: C, 37.2; H, 3.1; N, 21.7%); *δ*_H_ 1.22 (3H, t, ^3^*J*_HH_ 7.2, CH_3_), 3.51 (2H, q, ^3^*J*_HH_ 7.2, CH_2_); *δ*_C_ 14.8 (s, CH_3_), 37.3 (s, CH_2_), 113.45 (dd, ^2^*J*_CF_ 38, ^4^*J*_CF_ 8, C-5), 158.9 (dd, ^1^*J*_CF_ 216, ^3^*J*_CF_ 22, C-2), 162.7 (dd, ^3^*J*_CF_ 24, ^3^*J*_CF_ 6, C-4), 165.0 (dd, ^1^*J*_CF_ 263, ^3^*J*_CF_ 24, C-6); *δ*_F_ −46.14 (1F, s, C-6), −68.59 (1F, s, C-2); *m/z* (EI^+^) 193 ([M]^+^, 44%), 178 (62).

**5-Chloro-*N*,*N*-diethyl-2,6-difluoropyrimidin-4-amine (3c)**

A solution of 5-chloro-2,4,6-trifluoropyrimidine (1.01 g, 6 mmol), diethylamine (0.43 g, 6 mmol) and DIPEA (0.77 g, 6 mmol) in acetonitrile (100 cm^3^) was stirred at 0 °C for 2 h after which time ^19^F NMR indicated 100% conversion with the formation of 5-chloro-*N*,*N*-diethyl-2,6-difluoropyrimidin-4-amine (**3c**) and 5-chloro-*N*,*N*-diethyl-2,4-difluoropyrimidin-6-amine (**4c**) in a 5 : 1 ratio. The reaction solvent was evaporated and the crude product partitioned between DCM (3 x 40 cm^3^) and water (40 cm^3^). The organic layer was separated, dried (MgSO_4_) and evaporated to dryness to give a crude product containing **3c** and **4c** as a yellow oil (0.71 g). Column chromatography (silica, DCM : *n*-hexane, 1 : 2) gave 5-chloro-*N*,*N*-diethyl-2,6-difluoropyrimidin-4-amine (**3c**) (0.62 g, 47%) as a colourless oil; (Found: [MH]^+^ 222.0605; C_8_H_10_ClF_2_N_3_ requires: [MH]^+^ 222.0604); *δ*_H_ 1.25 (6H, t, ^3^*J*_HH_ 7.2, CH_3_), 3.68 (4H, q, ^3^*J*_HH_ 7.2, CH_2_); *δ*_C_ 13.9 (s, CH_3_), 45.2 (s, CH_2_), 92.5 (dd, ^2^*J*_CF_ 22, ^4^*J*_CF_ 9, C-5), 158.5 (dd, ^1^*J*_CF_ 190, ^3^*J*_CF_ 24, C-2), 161.5 (dd, ^3^*J*_CF_ 13, ^3^*J*_CF_ 5, C-4), 168.0 (dd, ^1^*J*_CF_ 233, ^3^*J*_CF_ 19, C-6); *δ*_F_ −47.81 (1F, s, C-6), −64.26 (1F, s, C-2); *m/z* (EI^+^) 221.0 ([M]^+^, 80%), 206 (86), 192 (88), 178 (98).

***N*-Benzyl-5-chloro-2,6-difluoropyrimidin-4-amine (3d)**

A solution of 5-chloro-2,4,6-trifluoropyrimidine (0.5 g, 3 mmol), benzylamine (0.32 g, 3 mmol) and DIPEA (0.39 g, 3 mmol) in acetonitrile (50 cm^3^) was stirred at 0 °C for 2 h after which time ^19^F NMR indicated 100% conversion with the formation of *N*-benzyl-5-chloro-2,6-difluoropyrimidin-4-amine (**3d**) (−45.80 and −67.84 ppm) and *N*-benzyl-5-chloro-4,6-difluoropyrimidin-2-amine (**4d**) (−48.09 ppm) in a 5 : 1 ratio. The reaction solvent was evaporated and the crude product partitioned between DCM (3 x 40 cm^3^) and water (40 cm^3^). The organic layer was separated, dried (MgSO_4_) and evaporated to dryness to give a crude product containing **3d** and **4d** as a yellow solid (0.54 g). Recrystallisation from *n*-hexane yielded *N*-benzyl-5-chloro-2,6-difluoropyrimidin-4-amine (**3d**) (0.31 g, 41%) as a white solid; mp 57–59 °C; IR (neat, *v*, cm^−1^): 3408, 3281, 2364, 2169, 1739, 1612, 1528, 1447, 1349, 1129, 695; (Found: C, 51.7; H, 3.1; N, 16.6; C_11_H_8_ClF_2_N_3_ requires: C, 51.7; H, 3.15; N, 16.4%); *δ*_H_ 4.74 (2H, d, ^2^*J*_HH_ 5.8, CH_2_), 7.39 (5H, m, Ar-H); *δ*_C_ 46.2 (s, CH_2_), 93.1 (dd, ^2^*J*_CF_ 21.4, ^4^*J*_CF_ 8.0, C-5), 128.1 (s, Ar-CH), 128.4 (s, Ar-CH), 129.2 (s, Ar-CH), 136.9 (s, Ar-CH), 159.3 (dd, ^1^*J*_CF_ 222, ^3^*J*_CF_ 22.1, C-2), 162.6 (dd, ^3^*J*_CF_ 13, ^3^*J*_CF_ 5.4, C-4), 164.5 (dd, ^1^*J*_CF_ 236.2, ^3^*J*_CF_ 18.7, C-6); *δ*_F_ −45.8 (1F, s, C-6), −67.9 (1F, s, C-2); *m/z* (EI^+^) 255 ([M]^+^, 40%), 218 (10), 178 (12).

***N*-*tert*-Butyl-5-chloro-2,6-difluoropyrimidin-4-amine (3e)**

A solution of 5-chloro-2,4,6-trifluoropyrimidine (0.5 g, 3 mmol), *tert*-butylamine (0.23 g, 3 mmol) and DIPEA (0.39 g, 3 mmol) in acetonitrile (50 cm^3^) was stirred at 0 °C for 2 h after which time ^19^F NMR indicated 100% conversion with the formation of *N*-*tert*-butyl-5-chloro-2,6-difluoropyrimidin-4-amine (**3e**) (−47.07 and −69.75 ppm) and *N*-*tert*-butyl-5-chloro-4,6-difluoropyrimidin-2-amine (**4e**) (−63.47 ppm) in a 3 : 1 ratio. The reaction solvent was evaporated and the crude product partitioned between DCM (3 x 40 cm^3^) and water (40 cm^3^). The organic layer was separated, dried (MgSO_4_) and evaporated to dryness to give a crude product containing **3e** and **4e** as a colourless solid (0.59 g). Recrystallisation from *n*-hexane yielded, *N*-*tert*-butyl-5-chloro-2,6-difluoropyrimidin-4-amine (**3e**) (0.32 g, 49%) as a colourless solid; mp 52–54 °C; (Found: C, 43.4; H, 4.6; N, 19.1; C_8_H_10_ClF_2_N_3_ requires: C, 43.4; H, 4.6; N, 19.0%); *δ*_H_ 1.12 (m, CH_3_); *δ*_C_ 28.9 (s, CH_3_), 54.0 (s, *C*CH_3_), 93.0 (dd, ^2^*J*_CF_ 21, ^4^*J*_CF_ 8, C-5), 159.8 (dd, ^1^*J*_CF_ 193, ^3^*J*_CF_ 23, C-2), 162.1 (dd, ^3^*J*_CF_ 20, ^3^*J*_CF_ 5, C-4), 164.3 (dd, ^1^*J*_CF_ 263, ^3^*J*_CF_ 19, C-6); *δ*_F_ −47.02 (1F, s, C-6), −69.70 (1F, s, C-2); *m/z* (EI^+^) 221 ([M]^+^, 38%), 206 (100).

**5-Chloro-2,4-difluoro-6-(piperidin-1-yl)pyrimidine (3f)**

A solution of 5-chloro-2,4,6-trifluoropyrimidine (0.5 g, 3 mmol), piperidine (0.25 g, 3 mmol) and DIPEA (0.39 g, 3 mmol) in acetonitrile (50 cm^3^) was stirred at 0 °C for 1h after which time ^19^F NMR indicated 100% conversion with the formation of 5-chloro-2,4-difluoro-6-(piperidin-1-yl)pyrimidine (**3f**) (−48.49 and −65.88 ppm) and 5-chloro-4,6-difluoro-2-(piperidin-1-yl)pyrimidine (**4f**) (−63.38 ppm) in a 3 : 1 ratio. The reaction solvent was evaporated and the crude product partitioned between DCM (3 x 40 cm^3^) and water (40 cm^3^). The organic layer was separated, dried (MgSO_4_) and evaporated to dryness to give a crude product containing **3f** and **4f** as a yellow oil (0.61 g). Column chromatography (silica, DCM : *n*-hexane, 10 : 1) gave 5-chloro-2,4-difluoro-6-(piperidin-1-yl)pyrimidine (**3f**) (0.34g, 49%) as a colourless oil; (Found: C, 46.4; H, 4.4; N, 17.9; C_9_H_10_ClF_2_N_3_ requires: C, 46.3; H, 4.3; N, 18.0%); *δ*_H_ 1.64 (6H, m, CH_2_), 3.73 (4H, m, CH_2_); *δ*_C_ 24.5 (s, CH_2_), 26.2 (s, CH_2_), 49.2 (s, CH_2_), 94.7 (dd, ^2^*J*_CF_ 21, ^4^*J*_CF_ 9, C-5), 158.3 (dd, ^1^*J*_CF_ 222, ^3^*J*_CF_ 22, C-2), 162.6 (dd, ^3^*J*_CF_ 13, ^3^*J*_CF_ 5, C-4), 164.5 (dd, ^1^*J*_CF_ 236, ^3^*J*_CF_ 19, C-6); *δ*_F_ −47.63 (1F, s, C-6), −64.17 (1F, s, C-2); *m/z* (EI^+^) 233 ([M]^+^, 70%), 204 (100).

***N*-(5-Chloro-2,6-difluoropyrimidin-4-yl)benzamidine (3g)**

A solution of 5-chloro-2,4,6-trifluoropyrimidine (1.1 g, 6.5 mmol), benzamidine hydrochloride (1.0 g, 6.5 mmol) and sodium hydrogencarbonate (0.55 g, 6.5 mmol) in acetonitrile (300 cm^3^) was stirred at room temperature for 24 h after which time ^19^F NMR indicated 100% conversion with the formation of *N*-(5-chloro-2,6-difluoropyrimidin-4-yl)benzamidine (**3g**) (−47.75 and −62.91 ppm) and *N*-(5-chloro-4,6-difluoropyrimidin-2-yl)benzamidine (**4g**) (−111.93 ppm) in a 40 : 1 ratio. The reaction solvent was evaporated and the crude product partitioned between DCM (3 x 40 cm^3^), water (40 cm^3^) and HCl (5 cm^3^). The organic layer was separated, dried (MgSO_4_) and evaporated to dryness to give a crude product containing **3g** and **4g** as a yellow solid (1.34 g). Recrystallisation from acetonitrile yielded *N*-(5-chloro-2,6-difluoropyrimidin-4-yl)benzamidine (**3g**) (1.21 g, 69%) as an off white solid; mp 164–165 °C; (Found: C, 48.9; H, 2.6; N, 20.7; C_11_H_7_ClF_2_N_4_ requires: C, 49.2; H, 2.6; N, 20.9%); *δ*_H_ (DMSO-*d*_6_) 7.50–7.61 (m, Ar-H); *δ*_C_ (DMSO-*d*_6_) 103.8 (dd, ^2^*J*_CF_ 14, ^4^*J*_CF_ 10, C-5), 129.4 (s, Ar-CH), 129.9 (s, Ar-CH) 133.5 (s, Ar-CH), 135.2 (s, Ar-CH), 158.2 (dd, ^1^*J*_CF_ 193, ^3^*J*_CF_ 23, C-6), 162.71 (s, C=N), 169.0 (dd, ^1^*J*_CF_ 224.9, ^3^*J*_CF_ 19.1, C-2), 170.33 (m, C-4); *δ*_F_ (DMSO-*d*_6_) −47.49 (1F, s, C-6), −66.06 (1F, s, C-2); *m/z* (EI^+^) 268 ([M]^+^, 12%), 233 (36). Crystals suitable for X-ray analysis were grown from acetonitrile.

*Crystal data for* ***3g***: C_11_H_7_ClF_2_N_4_, *M* = 268.66, monoclinic, space group *P*2_1_/*n*, *a* = 7.0749(2), *b* = 10.2084(3), *c* = 15.0143(5) Å, *β* = 91.76(1)°, *U* = 1083.87(6) Å^3^, F(000) = 544, *Z* = 4, *D*_c_ = 1.646 Mg m^−3^, *μ* = 0.365 mm^−1^ (Mo-Kα, *λ* = 0.71073 Å), *T* = 120.0(2) K. 11605 reflections were collected on a Bruker Proteum-M APEX diffractometer (ω-scan, 0.3°/frame) yielding 2878 unique data (R_merg_ = 0.0184). The structure was solved by direct method and refined by full-matrix least squares on F^2^ for all data using SHELXTL software. All non-hydrogen atoms were refined with anisotropic displacement parameters, H-atoms were located on the difference map and refined isotropically. Final wR_2_(F^2^) = 0.0972 for all data (191 refined parameters), conventional R (F) = 0.0340 for 2597 reflections with I ≥ 2σ, GOF = 1.025. Crystallographic data for the structure have been deposited with the Cambridge Crystallographic Data Centre as supplementary publication CCDC-681715.

^1^H NMR – ***N*-Benzyl-5-chloro-2,6-difluoropyrimidin-4-amine (3d)**


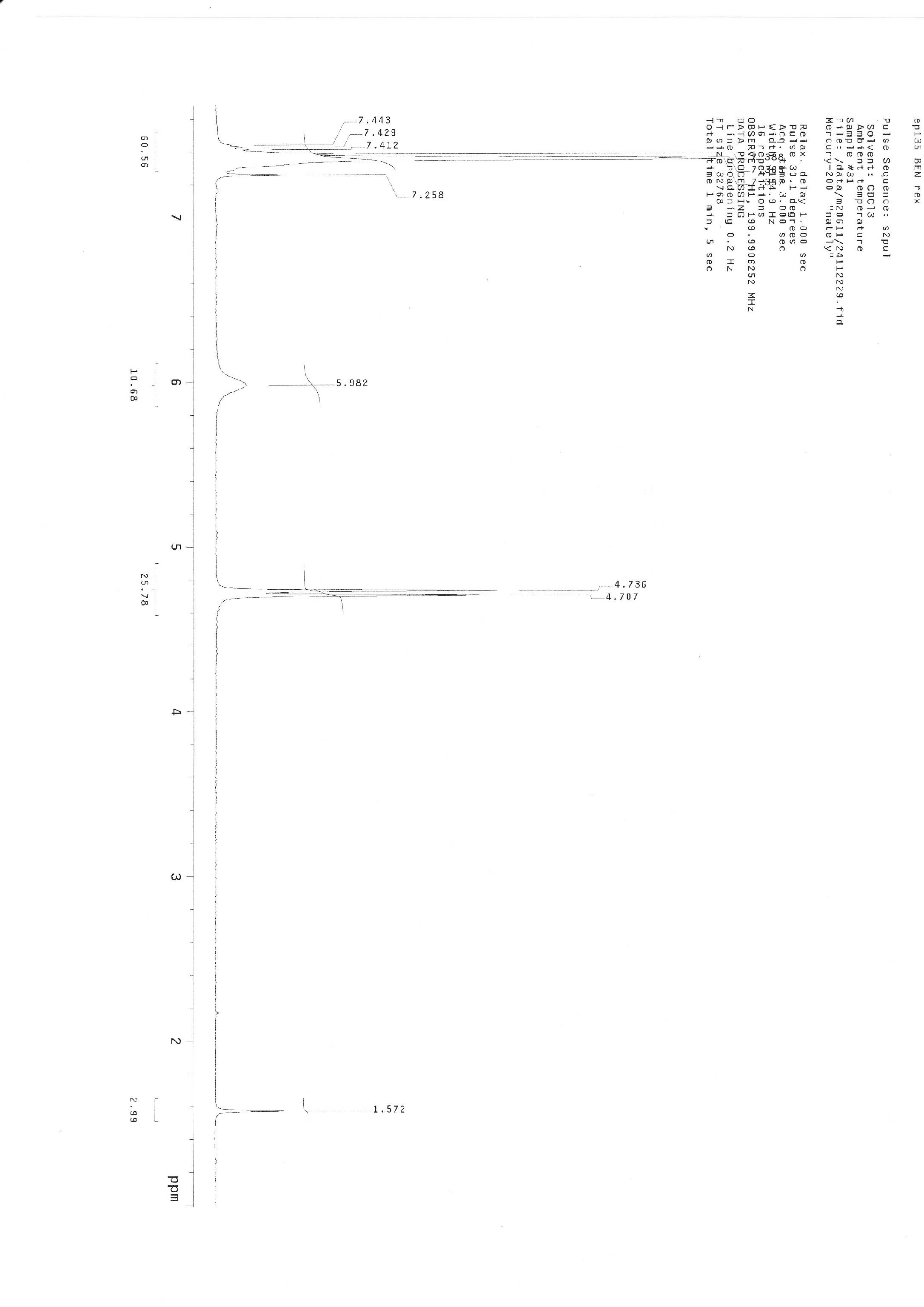


^19^F NMR – ***N*-Benzyl-5-chloro-2,6-difluoropyrimidin-4-amine (3d)**


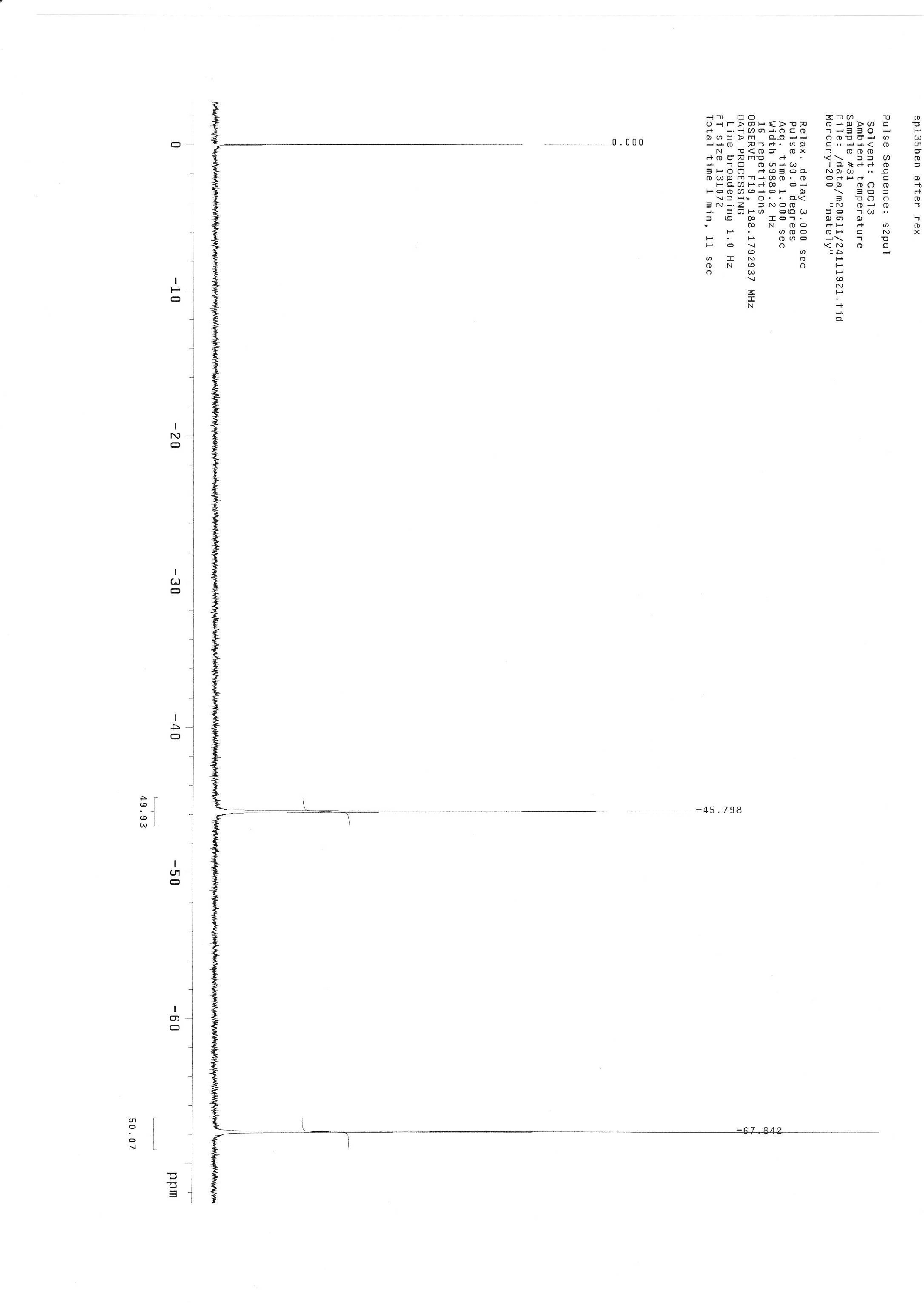


^13^C NMR – ***N*-Benzyl-5-chloro-2,6-difluoropyrimidin-4-amine (3d)**


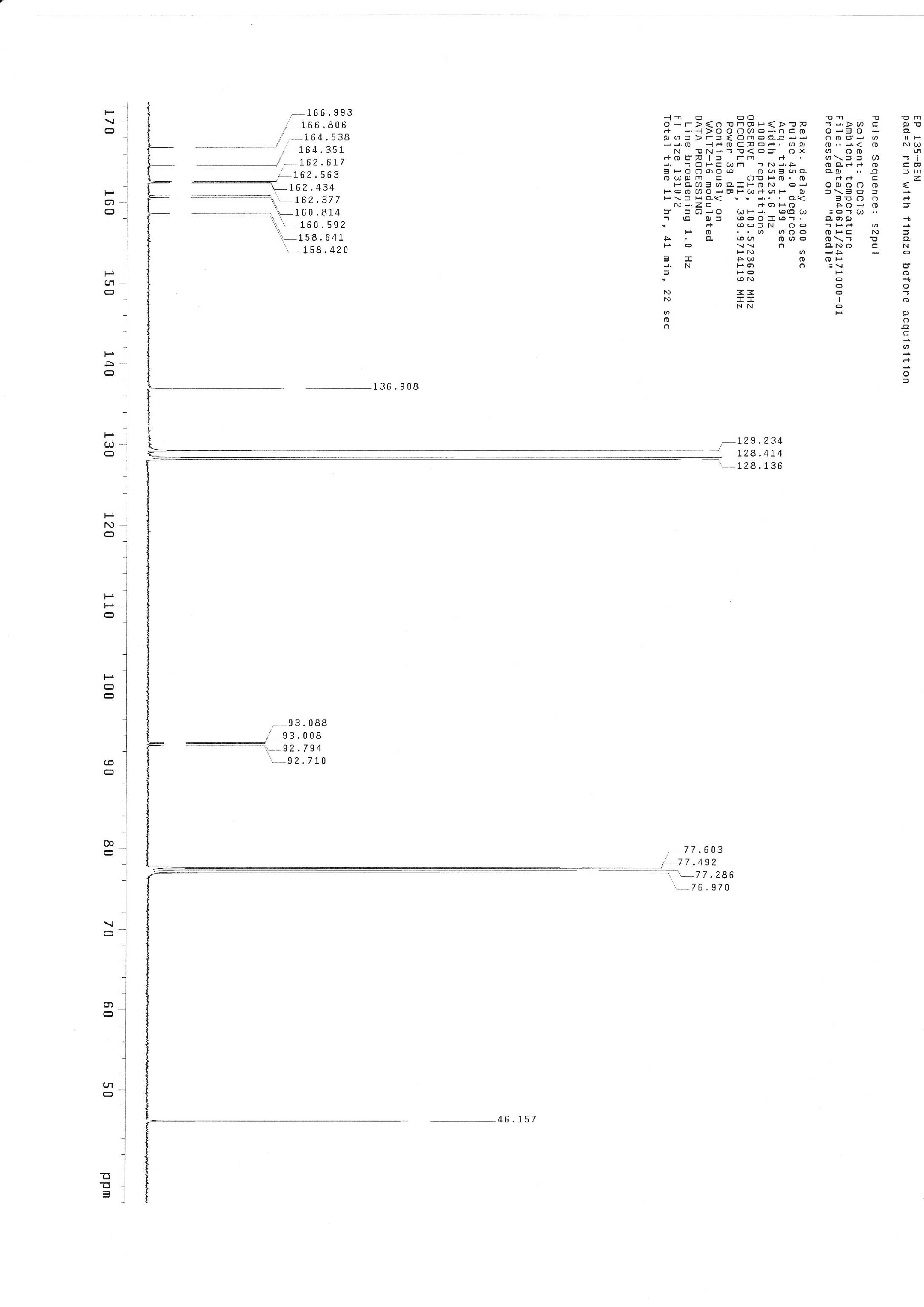

Supplement: File 1 — Experimental procedures and data. [file Beilstein_J_Org_Chem-04-22-s001.doc]
